# Emerging Roles of TWIK-1 Heterodimerization in the Brain

**DOI:** 10.3390/ijms19010051

**Published:** 2017-12-24

**Authors:** Chang-Hoon Cho, Eun Mi Hwang, Jae-Yong Park

**Affiliations:** 1 School of Biosystem and Biomedical Science, College of Health Science, Korea University, Seoul 136-703, Korea; chois007@korea.ac.kr; 2 Korea Institute of Science and Technology (KIST), Center for Functional Connectomics, Seoul 02792, Korea; 3 KHU-KIST Department of Converging Science and Technology, Kyung Hee University, Seoul 02447, Korea

**Keywords:** TWIK-1, K2P, astrocyte, brain, heterodimerization

## Abstract

Two-pore domain K^+^ (K2P) channels play essential roles in regulating resting membrane potential and cellular excitability. Although TWIK-1 (TWIK—tandem of pore domains in a weak inward rectifying K**^+^** channel) was the first identified member of the K2P channel family, it is only in recent years that the physiological roles of TWIK-1 have been studied in depth. A series of reports suggest that TWIK-1 may underlie diverse functions, such as intrinsic excitability of neurons, astrocytic passive conductance, and astrocytic glutamate release, as a homodimer or heterodimer with other K2P isotypes. Here, we summarize expression patterns and newly identified functions of TWIK-1 in the brain.

## 1. Introduction

Among the K^+^ channel families, two-pore domain potassium (K2P) channels are the most recently discovered. These K2P channels are major contributors to background potassium conductance, and control resting membrane potential and neuronal excitability [1]. In addition, these channels are modulated by a large number of physical and chemical stimuli, including temperature, membrane stretch, pH, polyunsaturated fatty acids, hormones, and neurotransmitters. Therefore, the 15 members of the K2P channel family play diverse physiological roles (e.g., adrenal gland development, thermal and mechanical nociception, and sensitivity to volatile anesthetics) [1]. K2P1 (KCNK1 or TWIK-1—tandem of pore domains in a weak inward rectifying K**^+^** channel) was initially cloned from a human kidney cDNA library [2]. However, due to the low or absent functional expression of K2P1 in heterologous expression systems, the physiological significance of TWIK-1 has remained enigmatic [3].

There have been two controversial hypotheses for the silencing mechanism of TWIK-1. One hypothesis is that TWIK-1 channels are present on or properly delivered onto the plasma membrane, but are silenced by a unique posttranslational modification, called sumoylation [3]. This was soon challenged by a report that TWIK-1 channels could not be sumoylated [4]. However, this “present but silent” hypothesis is still supported by a report that sumoylation of a single TWIK-1 subunit is sufficient to silence both TWIK-1 homodimeric and TWIK-1 heterodimeric channels [5,6]. The other hypothesis is that TWIK-1 channels are mainly located in intracellular compartments and are instead silenced by constitutive endocytosis and/or intracellular sequestration [7,8]. Because both hypotheses seem to be supported by convincing experimental data including mutagenesis at key residues, further investigations are needed to address the silencing mechanism of TWIK-1 channels.

Although the electrophysiological properties and functional roles of TWIK-1 are poorly understood due to nonmeasurable (or very small) TWIK-1 current in heterologous expression systems, the expression of TWIK-1 mRNA has been documented in various tissues, including the brain, heart, and kidney [2]. Mice deficient in TWIK-1 show defects in phosphate transport in the proximal tubule and water transport in the medullary collecting duct of the kidney, as well as deviations in the resting membrane potential of pancreatic β cells [9,10]. It has been also reported that TWIK-1 conducts inward leak Na^+^ currents during pathological hypokalemia in cardiomyocytes [11]. Interestingly, TWIK-1 knock-down zebrafish showed bradycardia and atrial dilation, which is consistent with atrial fibrillation identified in human TWIK-1 variants [12]. These previous observations strongly suggest the important physiological roles of TWIK-1 channels. Indeed, several recent studies have demonstrated unexpected roles of TWIK-1 in neurons and astrocytes. Here, we review emerging roles of TWIK-1 in the brain.

## 2. Distribution of TWIK-1 in the Brain

The original northern blot studies showed that TWIK-1 is highly expressed in the brain [13,14]. Subsequent in situ hybridization studies more clearly showed various expression patterns of TWIK-1 mRNA in the brain [15,16]. During development, the embryonic brain (at E17 and E19) has very low expression of TWIK-1 mRNA. In contrast, expression gradually increases after birth. In the early postnatal brain, its expression is mainly restricted to hippocampal neurons (dentate granule cells and pyramidal neurons). Its strong expression in dentate granule cells remains at a quite constant level throughout postnatal development. In hippocampal pyramidal neurons, it is enriched in CA3 relative to CA1 at P0 and maintains this graded expression pattern throughout development. In the rest of the forebrain (e.g., neocortex, thalamus, and striatum), TWIK-1 emerges gradually over the first postnatal weeks. In the reticular thalamus, TWIK-1’s expression is low at P7 and moderate at P14 and thereafter.

TWIK-1 is also expressed in cerebellar granule cells. Expression levels are low at P0; however, by P7, the external granule cell layer has moderate expression. By P14, when the external granule cell layer has greatly diminished in significance, there are still some TWIK-1 mRNAs present. In the internal granule cell layer, the TWIK-1 gene is not actually switched off between P14 and P28, and its mRNA is still present in the adult, but the level is decreased relative to the earlier expression in the same layer. Besides neuronal expression of TWIK-1, several studies reported that TWIK-1 is also expressed in astrocytes [17,18,19,20]. The expression of TWIK-1 mRNA in the astrocytes and neurons in various brain regions strongly suggests that TWIK-1 might be involved in diverse physiological roles in the brain.

## 3. Heterodimerization between TWIK-1 and Other K2P Isotypes

A previous biochemical study demonstrated that TWIK-1 subunits form homodimers in vitro, which are dissociated by treatment with β-mercaptoethanol, a reducing chemical agent. A disulfide bridge between the cysteines at residue 69 in the first extracellular loop links two subunits [21]. A recent report of the crystal structure also confirmed that TWIK-1 may assemble as a dimer via a disulfide bridge [22]. Although the disulfide bridge is critical for TWIK-1 homodimerization, it may not be required for homodimerization of other cysteine-containing K2P isotypes such as TWIK-2 and TWIK-related acid-sensitive K^+^ channel (TASK-2) [23,24]. In addition, TASK-1 and TASK-3, which lack the cysteine residue at these sites, can form functional channels as dimers [25,26]. Therefore, other unidentified mechanisms for dimerization of K2P channels seem possible.

In addition to the homodimerization of TWIK-1 via a disulfide bridge, it has been shown that TWIK-1 can heterodimerize with TASK-1 or TASK-3, using fluorescence resonance energy transfer (FRET) and immunoprecipitation in heterologously expressed chinese hamster ovary (CHO) cells, without providing any detailed molecular mechanism [5]. Because there is no cysteine residue in the first extracellular loop of TASK-1 and TASK-3 [26], these TWIK-1-containing heterodimers may not be formed via a cysteine disulfide bridge between these K2P isotypes. Recently, we reported that TWIK-1 and TWIK-related K^+^ channel (TREK-1) form heterodimers via a cysteine disulfide bridge in cultured astrocytes [20]. We also showed that TWIK-1 can form heterodimers with TREK-2 or TWIK-related arachidonic acid-stimulated K^+^ channel (TRAAK) via a disulfide bridge. These data strongly suggest that diverse heterodimers might be formed between different K2P isotypes, because the conserved cysteines in the first extracellular loop are present in several K2P isotypes (e.g., TWIK, TREK, and TWIK-related alkaline pH-activated K channel (TALK) subfamilies). The disulfide bridge is critical for the heterodimerization between TWIK-1 and TREK subfamily members (TWIK-1/TREK-1, TWIK-1/TREK-2, and TWIK-1/TRAAK heterodimers) [20]. Recent studies reporting heteromeric K2P channels (see Table 1) raised the possibility that many diverse K2P heterodimers might be assembled with or without the disulfide bridge, and their physiological functions will continue to be elucidated for years to come.

Because K2P heterodimeric channels are formed between two different isotypes, it is plausible that regulatory processes that are known to affect both subunits composing K2P heterodimers can also affect the activities of K2P heterodimers. For example, endocytosis or sumoylation could affect the activity of TWIK-1-containing heterodimers [3,6,8]. Indeed, TWIK-1/TREK-1 heterodimer channels appear to be actively regulated by endocytosis, although not by sumoylation [20]. In contrast, TWIK-1/TASK-1 and TWIK-1/TASK-3 heterodimers can be silenced by sumoylation [5]. Thus, new studies are required to better understand the regulatory mechanisms of various heterodimeric combinations of K2P channels.

## 4. Neuronal Function of TWIK-1

Although TWIK-1 is expressed in various types of neurons in the brain, its physiological roles in neurons are poorly understood. Only a few studies have shown neuronal functions of TWIK-1. Deng et al. reported that serotonin inhibits the excitability of stellate and pyramidal neurons in the entorhinal cortex by activating TWIK-1 [32]. The effects of serotonin are mediated via the serotonin 1A receptor (5-HT_1A_) and require the function of the Gα_i3_ subunit and protein kinase A. Because the entorhinal cortex acts as the gateway to the hippocampus, serotonin-mediated activation of TWIK-1 in neurons in the entorhinal cortex results in an inhibition of hippocampal circuits. It has not been determined whether TWIK-1, with other K2P isotypes in the entorhinal cortex, acts as a homodimer or a heterodimer.

In cerebellar granule neurons in which TWIK-1 is highly expressed [15,33], Plant et al. reported that TWIK-1 can form functional heterodimeric channels with TASK-1 or TASK-3 [5]. These heterodimer channels are regulated by sumoylation, halothane, or acids. Because the outward K^+^ current governs the response of cerebellar granule neurons to stimuli (e.g., volatile anesthetics and acids), these TWIK-1-containing heterodimer channels comprise the acid-sensitive and halothane-sensitive outward K^+^ currents in cerebellar granule neurons.

Our latest study also showed that TWIK-1 is expressed and localized mainly in the soma and proximal dendrites of dentate granule cells, rather than in distal dendrites or mossy fibers [34]. Gene silencing using a specific shRNA against TWIK-1 mRNA demonstrated that TWIK-1-mediated currents exhibit outwardly rectifying potassium currents and act as a contributor to the intrinsic excitability of the dentate granule cells. Due to the fact that TASK-3 is also highly expressed in these cells [5,16,35,36], it is possible that TWIK-1 functions as a heterodimer with TASK-3, resulting in the outward currents of TWIK-1/TASK-3 heterodimers.

## 5. TWIK-1 in Astrocytic Passive Conductance

Besides the neuronal expression of TWIK-1, astrocytes also express TWIK-1 [7,18,19,20]. Compared to other cell types, interestingly, astrocytes have an unusually leaky membrane with an extremely low membrane resistance. This property, termed passive conductance, typically exhibits a linear current–voltage relationship, which implies the predominant expression of K^+^ channels that differ from conventional voltage-gated or leak K^+^ channels. However, the molecules responsible for this conductance have not been identified.

Zhou et al. previously suggested that TWIK-1 and TREK-1 independently contribute to the passive conductance of astrocytes, based on comparative studies between biophysical and pharmacological properties of the passive conductance in astrocytes and of currents mediated by either a cloned TWIK-1 mutant (TWIK-1·K274E) or cloned TREK-1 channels in heterologous expression systems [18]. Interestingly, in a subsequent study, the same group concluded that the overall astrocytic passive conductance is not significantly altered in TWIK-1 knockout mice, although their experimental data showed that the astrocytic membrane properties were altered (membrane potential and rectification index of passive conductance) [6]. In contrast, our study showed that the passive conductance was dramatically reduced in astrocytes when endogenous TWIK-1 channels were ablated by TWIK-1-specific shRNA [20]. Accompanying biochemical and electrophysiological data showed that TWIK-1 forms a heterodimeric channel with TREK-1, and this TWIK-1/TREK-1 heterodimer mediates the passive conductance in astrocytes. Due to these conflicting reports, further investigations will be required to determine the molecular identities of the passive conductance and the physiological role of TWIK-1 in astrocytes [7,20].

## 6. TWIK-1 in Astrocytic Glutamate Release

Astrocytes release glutamate upon activation of various GPCRs (G-protein coupled receptors) to modulate various types of synaptic transmissions and plasticity [37]. Although it is clear that neurons release glutamate via Ca^2+^-dependent exocytosis, the molecular mechanism of glutamate release from astrocytes is poorly understood. We demonstrated that TREK-1 and Bestrophin-1 (Best1), a Ca^2+^-activated Cl^−^ channel, mediate fast and slow glutamate release respectively in astrocytes upon GPCR activation [37]. In this study, we also confirmed that TWIK-1/TREK-1 heterodimers act as the precise molecular entity responsible for the fast mode of glutamate release upon activation of G_αi_-coupled GPCRs (CB1 in particular) in astrocytes [20,37]. This TWIK-1/TREK-1 heterodimer is one of a few known cases of G_βγ_ binding to ion channels (e.g., inwardly rectifying K channels (GIRKs), some voltage-gated Ca^2+^ channels, and transient receptor potential melastatin channels [38,39,40,41,42,43].

Yeast two-hybrid screening data indicated that TWIK-1 can interact with the G_γ_ subunit directly through their cytosolic N- and C-termini, like TREK-1 [20]. In this study, the concatenated TWIK-1–TREK-1 channel was permeable to glutamate and potassium ions upon G_βγ_ application. These data imply that the pore region of the TWIK-1/TREK-1 heterodimer might be altered upon G_βγ_ application. Although the precise nature and characteristics of the glutamate-permeable pore within the TWIK-1/TREK-1 heterodimer are unknown, the existence of unconventional pore domains (GLG or GFG motif) within TWIK-1 and TREK-1 may contribute to the mechanism underlying the dilation of the pore. To understand how TWIK-1/TREK-1 heterodimers can be transformed to glutamate-permeable channels upon GPCR activation, further studies are needed.

## 7. Implication of TWIK-1 in the central nervous system (CNS)/Brain Diseases

In a whole-genome microarray study using monozygotic twins discordant, TWIK-1 expression was increased more than two-fold in lymphoblastoid cells from people with bipolar disorders [44]. Except for this study, there is no report relating TWIK-1 with CNS diseases. However, the link between TWIK-1 and GPCR activation suggests that TWIK-1 could be involved in brain diseases. For example, activation of PAR1 (protease-activated receptor-1) in astrocytes by high levels of thrombin at the site of a brain lesion could activate glutamate release through the TWIK-1/TREK-1 heterodimer [20,37,39,40,44,45]. It will be also intriguing to see whether the neuroprotective effect of targeting 5-HT1a receptors in striatal astrocytes is caused by inhibition of TWIK-1/TREK-1 channel activity in Parkinson’s disease [8,46]. In addition, targeting mGlu3 receptors in astrocytes to ameliorate neurotoxicity of amyloid β (Aβ) in Alzheimer’s disease may act through TWIK-1/TREK-1 heterodimeric channels [47].

## 8. Perspectives

**Figure 1:**
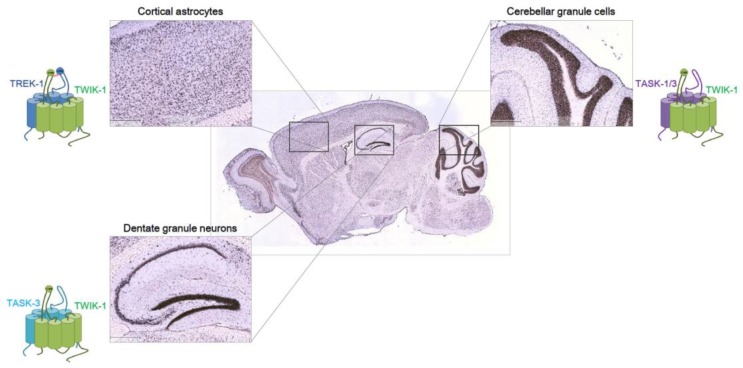
Heterodimeric K2Ps in the brain. Expression pattern of mouse TWIK-1 (TWIK—tandem of pore domains in a weak inward rectifying K**^+^** channel) from Allen Brain Atlas database. In situ hybridization of TWIK-1 is shown in a sagittal section of mouse brain at P56. Shown are higher magnification of cortex (**top left**), hippocampus (**bottom left**), cerebellum (**top right**), and medulla (**bottom right**) from serial sections. Scale bar: 419 μm. Note that TWIK-1 can function as either a homodimeric or heterodimeric channel. Based on the reports that TWIK-1 heterodimeric channels function in three brain regions [20,26,35], we believe TWIK-1 acts as heterodimeric channels with other K2P partners in other brain regions. Functional heterodimerization of TREK-1/TWIK-1 in cortical astrocytes [20], TWIK-1/TASK-1/-3 in cerebellar granule cells [26], and TWIK-1/TASK-3 [35] in dentate granule neurons have been reported.

The physiological roles of TWIK-1, the first identified K2P channel, have not been characterized. A series of pathfinding studies showed that TWIK-1 displays diverse functions such as intrinsic excitability of neurons, astrocytic passive conductance, and astrocytic glutamate release in the brain. Based on the distinct expression patterns of TWIK-1 in the brain and accumulating evidence for TWIK-1-containing heterodimers (Figure 1), it is possible that different combinations of TWIK-1-containing heterodimers exist. Furthermore, signaling pathways for the regulation of TWIK-1 heterodimeric channels is important. Upon TWIK-1 heterodimerization with other K2P channels, we believe that TWIK-1 modulators involved in sumoylation, endocytosis, and GPCR-mediated signaling of TWIK-1 regulate TWIK-1 heterodimeric channels [5,20]. We also believe that modulators of TWIK-1 partners regulate TWIK-1 heterodimeric channels. For example, a neuropeptide, neurotensin, inhibits TASK-3 [48], and we have observed that neurotensin also inhibits TWIK-1/TASK-3 heterodimer channels (data not shown) [49]. We expect that further research will uncover numerous and diverse functions of TWIK-1 in the brain.

## Figures and Tables

**Table 1:** Heterodimers of two-pore domain potassium (K2P) channels.

K2P Dimer	Validation		Disulfide Bond	Physiological Function	Ref.
	In Vitro	In Vivo			
TASK-1/TASK-3		Co-IP	N.D.	The heterodimeric channels mediate the pH and isoflurane-sensitive K^+^ currents in hypoglossal motoneurons.	[26]
TWIK-1/TASK-1 (or TASK-3)	FRET, Co-IP		N.D.	The heterodimeric channels comprise the acid-sensitive K^+^ currents and response to halothane in cerebellar granule cells.	[5]
TWIK-1/TREK-1	Co-IP, BiFC, MY2H	Co-IP, PLA	Dependent (TWIK-1 C69/ TREK-1 C93)	The heterodimeric channels mediate passive conductance and fast glutamate release in cortical astrocytes.	[20]
TWIK-1/TREK-2	Co-IP		Dependent (TWIK-1 C69)	N.D.	[20]
TWIK-1/TRAAK	Co-IP		Dependent (TWIK-1 C69)	N.D.	[20]
THIK-1/THIK-2	FRET, PLA		N.D.	N.D.	[27]
TRAAK/TREK-1 (or TREK-2)	SiMPull TIRF imaging FRET PLA		N.D.	N.D.	[28,29]
TREK-1/TREK-2	Co-IP	Single channel recording	N.D.	N.D.	[30]
TASK-1/TALK-2	BiFC, FRET, Co-IP	TIRF imaging	N.D.	N.D.	[31]

FRET: Fluorescence resonance energy transfer, Co-IP: Co-immunoprecipitation, BiFC: Bimolecular fluorescence complementation, MY2H: membrane yeast two-hybrid, SiMPull: single-molecule pull-down, TIRF: Total internal reflection fluorescence, PLA: Proximity ligation assay, N.D. = not determined.

## Abbreviations

TWIK	tandem of pore domains in a weak inward rectifying K^+^ channel
TREK	TWIK-related K^+^ channel
TASK	TWIK-related acid-sensitive K^+^ channel
TRAAK	TWIK-related arachidonic acid-stimulated K^+^ channel
TALK	TWIK-related alkaline pH-activated K channel
THIK	tandem pore domain halothane-inhibited K^+^ channel
CHO	chinese hamster ovary
5-HT_1A_	serotonin 1A receptor
CNS	central nervous system
Aβ	amyloid β
